# A Spontaneous Spinal Epidural Hematoma Secondary to Long-Term Low-Dose Aspirin and Clopidogrel Use: A Case Report

**DOI:** 10.7759/cureus.34537

**Published:** 2023-02-02

**Authors:** Mohammad Abu-Abaa, Omar Jumaah, Aliaa Mousa, Ghassan Al-Qaysi

**Affiliations:** 1 Internal Medicine, Capital Health Regional Medical Center, Trenton, USA

**Keywords:** back pain, paralysis, clopidogrel, aspirin, epidural hematoma

## Abstract

The association between antiplatelet agents such as aspirin, clopidogrel, and ticlopidine and spontaneous spinal epidural hematoma is based on multiple case reports in the literature. Here, we present the case of a 76-year-old male patient who presented with acute low back pain associated with sudden-onset paralysis of the lower extremities. His past medical history was remarkable for coronary artery disease with a stent placement history on dual antiplatelet therapy including low-dose aspirin and clopidogrel. An extensive posterior thoracolumbar epidural hematoma was seen on the imaging test, and rapid clinical improvement was evident early during his presentation. This prompted a conservative approach that led to complete neurological recovery. This case is in line with limited English-language literature evidence that suggests a possible association between spontaneous spinal epidural hematoma and antiplatelet agents. We aim to enhance clinicians’ awareness of this clinical entity, association, presentation, and management.

## Introduction

Spontaneous spinal epidural hematoma (SSEH) is a rare but devastating entity. Although the pathogenesis remains unclear, it is plausible that SSEH is a result of the rupture of epidural veins, which are valveless and susceptible to rupture as these veins are not protected from fluctuations in pressure in the abdomen and thorax [1]. This is supported by the observation that the most common location of SSEH is the cervicothoracic or thoracolumbar junction mainly posteriorly where epidural veins are abundant [2]. Three antiplatelet agents have been suggested in association with SSEH, including aspirin, clopidogrel, and ticlopidine [3,4]. Lab tests usually cannot rule out platelet dysfunction [3]. The causal relationship between SSEH and antiplatelet agents is based on a limited number of case reports. This case aims to support such limited evidence. This case supports the current recommendation that rapid neurological improvement in SSEH should prompt conservative management.

## Case presentation

A 76-year-old male patient presented to the emergency department (ED) with sudden, severe, bilateral lower extremity weakness and loss of sensation associated with sharp low back pain and abdominal pain. He reported severe weakness starting shortly after taking a bath in the morning to the point of laying down on the floor and not being able to get up. The pain was located in the lower back and radiated to the lower abdomen. His past medical history was remarkable for coronary artery disease on aspirin 81 mg daily and clopidogrel 75 mg daily for coronary stents five years ago and hyperlipidemia. In the ED, vital signs included a temperature of 36.5°C, heart rate of 63 beats per minute, respiratory rate of 18 cycles per minute, blood pressure of 150/90 mmHg, and SpO_2_ of 98% on room air. A physical examination showed mild periumbilical tenderness with diminished sensation to light touch below the level of the umbilicus. Hip flexion muscle power was 2/5 bilaterally, knee flexion and extension were 3/5 bilaterally, and plantar flexion was 2/5 bilaterally with hyporeflexia at the knees and ankles bilaterally. T8 sensory level was evident. Basic labs were unremarkable. CT angiography of the chest was remarkable only for a 4.1-cm fusiform ascending aortic aneurysm with no dissection. CT angiography of the abdomen and pelvis with/without contrast was also unremarkable. CT of the head, cervical, thoracic, and lumbar spine was unremarkable. During evaluation in ED, the patient was able to report a slight improvement in lower limb weakness but developed urinary retention that prompted the insertion of a Foley catheter.

**Figure 1:**
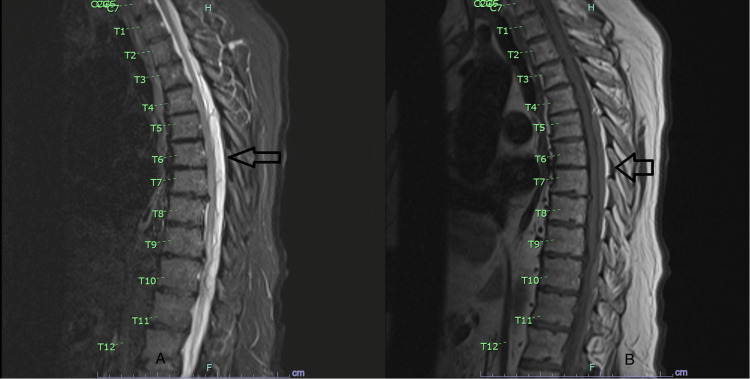
MRI short tau inversion recovery and T1 of the thoracic spine. (A) An MRI short tau inversion recovery sequence showing extensive posterior thoracic epidural hematoma. (B) An MRI T1 sequence showing similar findings with shortening and subtle enhancement (arrows).

**Figure 2:**
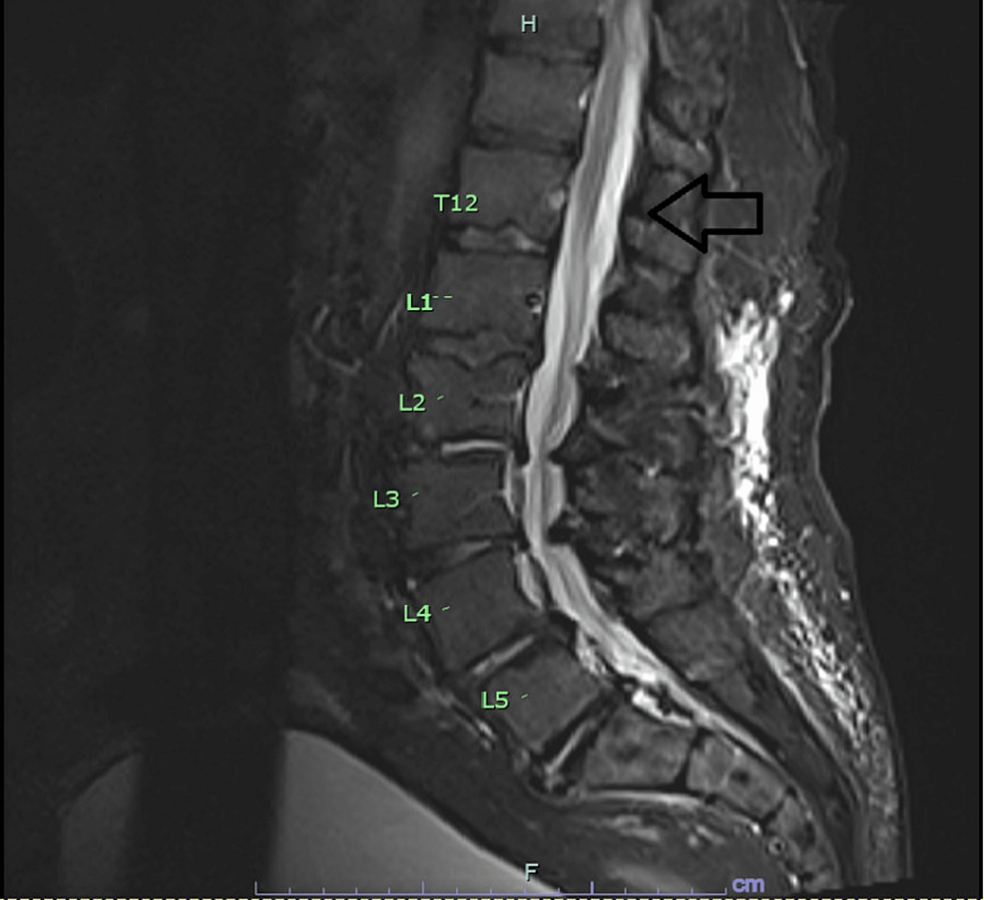
MRI of the lumbar spine showing epidural hematoma (arrow).

MRI of the thoracic and lumbar spine showed fluid collection throughout the posterior thoracic spinal canal extending from T2-3 to L1 level with slight T1 shortening and subtle enhancement about 8-mm in thickness suggestive of epidural hematoma with no evidence of fistula (Figures 1, 2). An absence of spinal arteriovenous fistula and malformation was also confirmed by magnetic resonance angiography of the spine. Clopidogrel and aspirin were both held. A coagulation profile including prothrombin time, partial thromboplastin time, international normalized ratio, and bleeding time were within the normal range. Given the rapid improvement in the symptoms, no surgical intervention was pursued, and watchful observation was attempted. Three days into his hospitalization, his neurological examination showed improved muscle power of the lower extremities to 4/5 bilaterally. No further evidence of sensory deficit or urinary retention was noted. This improvement allowed for discharge to a medical rehabilitation facility after four days of hospitalization.

**Figure 3:**
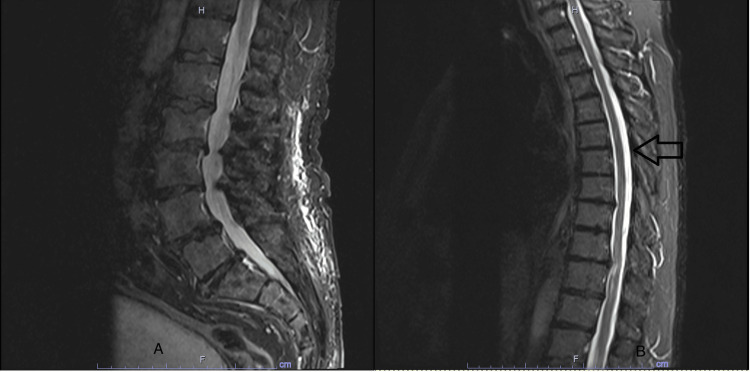
MRI of the thoracic and lumbar spine. (A) MRI of the lumbar spine and (B) MRI of the thoracic spine showing improvement in the epidural hematoma (arrows).

At a follow-up one month later, the patient demonstrated near-complete clinical neurological recovery. A repeat MRI of the spine showed that epidural hematoma had improved (Figure 3). A diagnostic spinal angiogram was also negative. Two months post-hematoma, no antiplatelet agent was restarted.

## Discussion

SSEH is a rare entity with an estimated incidence of 0.1% per 100,000. This accounts for less than 1% of spinal epidural lesions [5]. It is most commonly idiopathic but has been seen in patients using anticoagulants, with coagulopathies, tumors, pregnancy, infections, and vascular malformations [6]. However, SSEH related to antiplatelet agents is very rare. Only a few cases of SSEH related to low-dose aspirin have been reported [7-9]. A literature review also showed only four cases of possible clopidogrel association with SSEH [10-13]. Similar cases of SSEH in patients on both aspirin and clopidogrel have also been reported rarely [14]. To our knowledge, no previous studies provided an estimate of the risk of SSEH in association with aspirin and clopidogrel.

In our patient, except for the use of low-dose aspirin and clopidogrel, no history of similar risk factors was evident. No history of trauma or use of anticoagulant was reported. Furthermore, there was no evidence of aneurysm, vascular malformation, coagulopathy, malignancy, or infection during the extensive workup. In addition, the relatively long interval duration between the initiation of aspirin and clopidogrel and the onset of SSEH does not rule out the association as a previously reported case had intervals as long as 10 years prior to SSEH [3]. Therefore, ruling out the role of antiplatelet agents in inducing SSEH is not possible. Normal bleeding time does not rule out the association of aspirin with SSEH as a significant number of patients with low-dose aspirin-related intracranial hemorrhage have normal bleeding time [15].

SSEH has a wide spectrum of clinical presentation depending on the degree of spinal cord compression and its extent. It can range from radiculopathy and paraplegia to complete quadriplegia [6]. The typical presentation is acute severe back pain or neck pain radiating to the corresponding dermatome and associated with or followed by signs and symptoms of nerve or spinal cord compression [16]. Ascending paralysis mimicking Guillain-Barre syndrome or cauda equina syndrome or isolated sensory deficit has also been reported [17].

The treatment of choice is immediate surgical decompression. However, mild neurological involvement and/or rapid neurological recovery are indications for conservative management [5,18]. There is debate regarding the management of coagulopathy-associated SSEH. Some have recommended replacement therapy, while others have suggested that coagulopathy prolongs the time that bleeding remains liquid which allows for even distribution along the epidural space and successful management by a conservative approach [19,20]. The current recommendation is a short period of serial neurological assessment, and if no neurological improvement is evident, then surgical decompression is indicated [21]. There is no clear recommendation for restarting antiplatelet therapy in SSEH cases. The limitation of our study is that a causal relationship is difficult to conclude based on a single case. Thus, the association between SSEH and antiplatelet agents needs further research to investigate the strength of a causal relationship.

## Conclusions

Although rare, SSEH should always be suspected when a patient on an antiplatelet agent presents with a new-onset neurological deficit. The typical presentation is acute severe back pain followed by signs and symptoms of spinal cord compression. Although surgical decompression is the preferred approach in most reported cases, this case demonstrates that a conservative approach can lead to full neurological recovery. A rapid neurological improvement, as was seen in this case, should prompt conservative management.
